# Supplementary material for: The Preparation and Evaluation of Cyanocobalamin Mucoadhesive Sublingual Tablets
Source: Pharmaceuticals (Basel). 2023 Oct 4;16(10):1412. doi: 10.3390/ph16101412 (PMC10610133; doi:10.3390/ph16101412)
Supplement: Supplementary file 1 [file pharmaceuticals-16-01412-s001.zip › pharmaceuticals-2593194-supplementary.pdf]

The Preparation and Evaluation of Cyanocobalamin Mucoadhesive Sublingual Tablets

Anwar Ma'ali, Hani Naseef *, Moammal Qurt, Abdallah Damin Abukhalil, Abdullah K. Rabba and Israr Sabri

Pharmacy Department, Faculty of Pharmacy, Nursing and Health Professions, Birzeit University,
Ramallah P.O. Box 14, Palestine; anwarmaali95@gmail.com (A.M.); mqurt@birzeit.edu (M.Q.);
adkhalil@birzeit.edu (A.D.A.); arabba@birzeit.edu (A.K.R.); isabri@birzeit.edu (I.S.)
* Correspondence: hshtaya@birzeit.edu; Tel.: +970-598903449

Supplementary materials

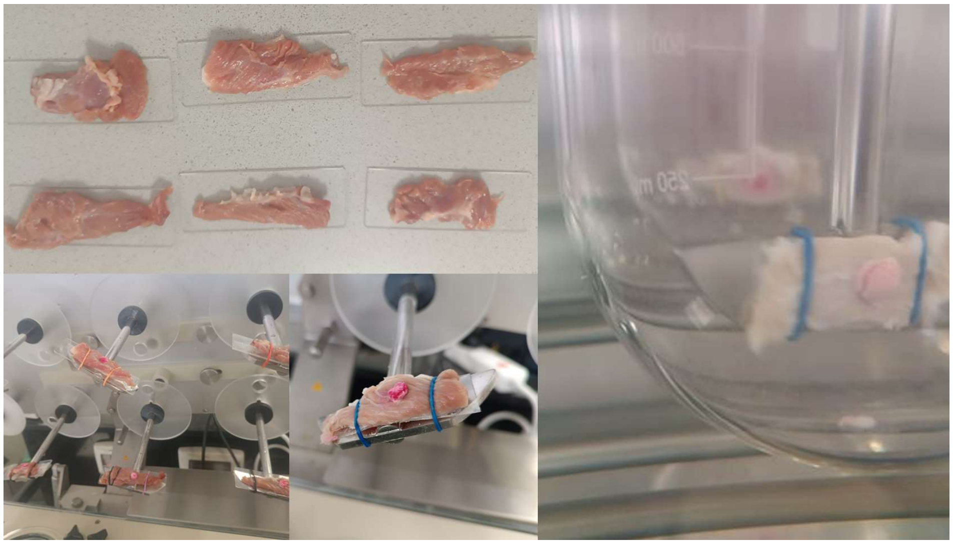

Figure S1. Sublingual mucosa fixed to the slide for mucoadhesive residence time evaluation.

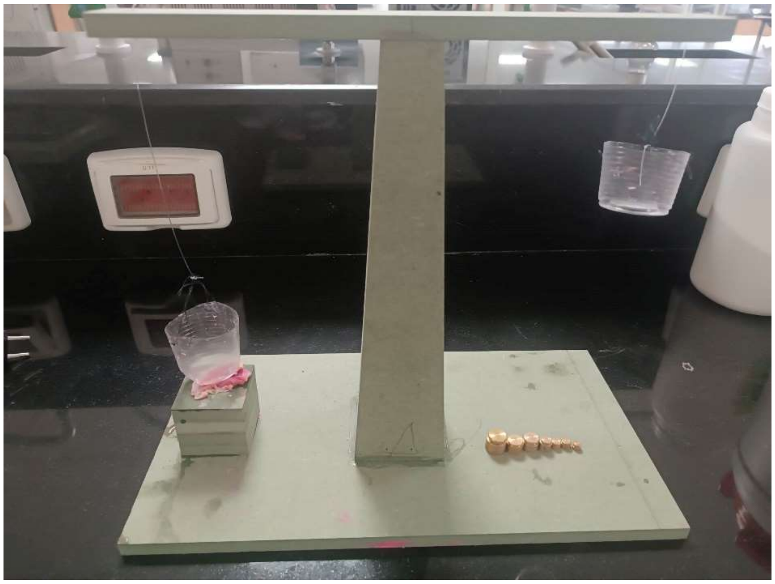

Figure S2. The balance model that was used to check the tablet's mucoadhesive strength

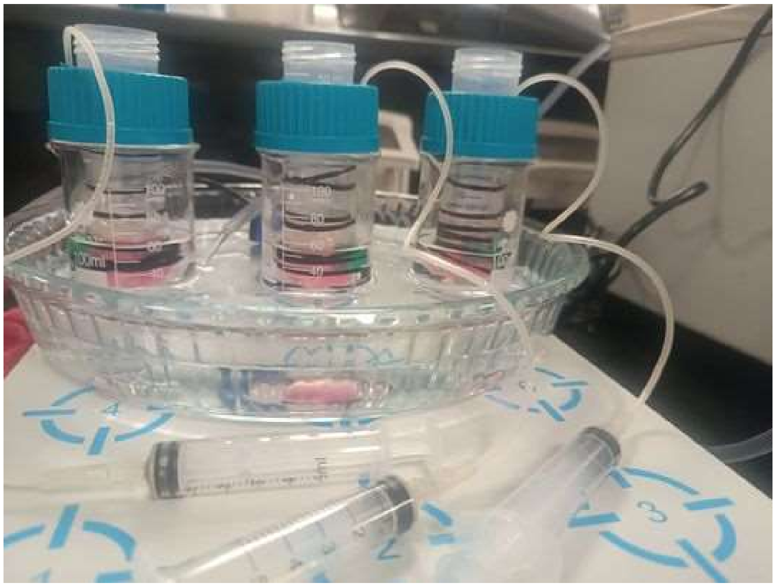

Figure S3. A model system for drug release test

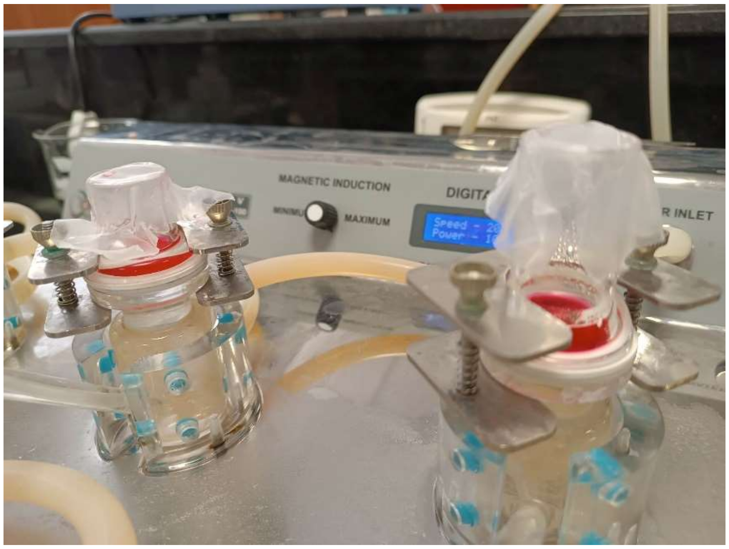

Figure S4. The Franz diffusion cell used in the cyanocobalamin permeation test.
